# AI Will Offer New Opportunities for Transplantation: Are We Ready?

**DOI:** 10.1097/TXD.0000000000001820

**Published:** 2025-05-29

**Authors:** Umberto Maggiore, Jamil Azzi, Leonardo V. Riella, Paolo Cravedi

**Affiliations:** 1 UO Nefrologia, Dipartimento di Medicina e Chirurgia, Università di Parma, Parma, Italy.; 2 Transplantation Research Cenyounter, Renal Division, Brigham and Women’s Hospital and Children’s Hospital, Boston, MA.; 3 Department of Surgery, Center for Transplantation Sciences, Massachusetts General Hospital, Harvard Medical School, Boston, MA.; 4 Department of Medicine, Translational Transplant Research Center, Icahn School of Medicine at Mount Sinai, New York, NY.

For decades, a narrow pipeline of antirejection molecules has contributed to the limited advancements in long-term outcomes of organ transplant recipients.^1^ This may soon change. The rapid advancement of artificial intelligence (AI) is expected to significantly expand the development of novel agents targeting the alloimmune response.^2^ Already used to accelerate drug discovery, AI aids in identifying new targets, optimizing molecules, and repurposing drugs. By analyzing large-scale omics data sets, AI-driven models can predict drug-target interactions and generate potential therapeutic molecules with enhanced specificity and efficacy. For example, DSP-1181, an AI-designed drug-targeting obsessive-compulsive disorder, progressed from initial screening to phase I clinical trials in <12 mo.^3^ Are we ready?

Most transplant trials are conducted with few patients, translating to insufficient statistical power, or are performed as single-arm trials that make it difficult to compare against other therapeutic options. With the rise in precision medicine, these challenges have been amplified. Although the importance of well-conducted randomized trials and the inclusion of placebo or standard-of-care arms remains unquestioned, the development of data sources and analytic methodologies to construct synthetic control arms from external datasets has advanced significantly in recent years, particularly in oncology.^4^ Synthetic control arms provide several benefits, such as reduced budgets, limited site and patient burden, and potentially shorter execution timelines while also having the potential to eliminate ethical concerns regarding placebo control groups.^2^ The use of digital twins for in silico randomized clinical trials represents a further significant advancement in leveraging generative AI to evaluate new treatments efficiently.^5^ Unlike synthetic control arms, which rely on external data sources such as historical clinical trials and real-world data, digital twins incorporate model-based predictions of individual patient outcomes as if they had been in the control group.^5^ Although these approaches hold great promise, transplantation faces unique challenges, such as recipient heterogeneity and variable immunosuppressive regimens across centers, which must be carefully addressed when implementing AI-driven clinical trial designs.

The transplant community should proactively develop innovative strategies to navigate the evolving landscape of new therapies, requiring both investment and regulatory reforms for effective and safe clinical translation. As the Food and Drug Administration and the European Medicines Agency explore the integration of synthetic data into the drug approval process, transplant investigators must actively engage in shaping these discussions to ensure that AI-driven methodologies align with the unique needs of the field.^5,6^ Delayed action could hinder the integration of emerging therapies.

Expanding the discussion on AI in transplantation is essential, engaging all key stakeholders, including the transplant community, regulatory agencies, industry leaders, and policymakers. Future transplant meetings and journal forums should prioritize this dialogue to ensure a collaborative and adaptive approach. AI is poised to revolutionize medicine, but the actual impact on patients’ health will largely depend on our level of preparedness to embrace these opportunities and drive meaningful changes in trial design, regulatory frameworks, and pharmaceutical engagement.

## References

[1] Poggio ED Augustine JJ Arrigain S. Long-term kidney transplant graft survival—making progress when most needed. Am J Transplant. 2021;21:2824–2832. 33346917 10.1111/ajt.16463

[2] Zhang K Yang X Wang Y. Artificial intelligence in drug development. Nat Med. 2025;31:45–59. 39833407 10.1038/s41591-024-03434-4

[3] Burki T. A new paradigm for drug development. Lancet Digit Health. 2020;2:e226–e227. 32373787 10.1016/S2589-7500(20)30088-1 PMC7194950

[4] Thorlund K Dron L Park JJH. Synthetic and external controls in clinical trials—a primer for researchers. Clin Epidemiol. 2020;12:457–467. 32440224 10.2147/CLEP.S242097 PMC7218288

[5] Katsoulakis E Wang Q Wu H. Digital twins for health: a scoping review. NPJ Digit Med. 2024;7:77. 38519626 10.1038/s41746-024-01073-0 PMC10960047

[6] Askin S Burkhalter D Calado G. Artificial intelligence applied to clinical trials: opportunities and challenges. Health Technol (Berl). 2023;13:203–213. 36923325 10.1007/s12553-023-00738-2 PMC9974218

